# The Impact of Household Size on Lexical Typicality: An Early Link Between Language and Social Cognition?

**DOI:** 10.3389/fpsyg.2018.01445

**Published:** 2018-08-14

**Authors:** Julien Mayor, Natalia Arias-Trejo, Elda A. Alva

**Affiliations:** ^1^Department of Psychology, University of Oslo, Oslo, Norway; ^2^Facultad de Psicología, Universidad Nacional Autónoma de México, Mexico City, Mexico

**Keywords:** language, social communication, infants, children, word learning, communicative development inventories

## Abstract

To communicate successfully, speakers need to use words that are understood by their listeners; they thus need to understand that others have vocabularies different than their own. A key question is whether this social cognition skill is already present in infancy, and whether it can have an impact on early language production. Analysis of the vocabularies of 550 Mexican children revealed that, at 24 months of age, but not at 18 nor at 30 months of age, those who were raised in households with larger numbers of children had more stereotypical vocabularies than those with fewer children. This finding is discussed in light of the hypothesis that communicative pressure may shape early word production; it suggests that bidirectional effects between social cognition and language acquisition are present at 2 years of age.

## Introduction

Language has evolved to facilitate communication between human beings. For a communicative act to be successful, an alignment is needed between a speaker’s utterances and a listener’s receptive lexicon. For example, adults addressing a young child typically attune their utterances to fit the child’s limited lexicon (Baumwell et al., 1997). This behavior implies social cognition skills, in particular the ability to recognize limitations in other people’s language. However, infants and young children embark on the process of learning a language with immature social cognition skills. There is considerable evidence that language plays a prominent role in shaping social cognition in general, and in establishing mature Theory of Mind in particular (Ruffman et al., 2002; Astington and Baird, 2005; Milligan et al., 2007). Conversely, early word learning is embedded in social contexts, where shared attention provides support for reducing the hypothesis space in a naming event (Quine, 1960; Tomasello and Farrar, 1986). An understanding of social cues is instrumental in driving language development. We can thus ask when children recognize that different interlocutors have distinct vocabulary sizes and compositions, and when they can use these social cognition skills to adjust their communicative acts to the differing language skills of others.

Several methods can be employed to address these questions. One is to design experimental tasks to compare a child’s use of language addressing adults with that addressing other children. This approach allows for an online measure of language fine-tuning in addressing different speakers; one such study has revealed that 4-year-old children adapt their speech when speaking to younger children (Shatz and Gelman, 1973).

Another technique is to evaluate whether a child’s lexicon may be shaped by understanding that not all speakers possess large vocabularies. This measurement can be performed by evaluating the overlap between individual lexicons: an individual productive lexicon aligning with many other lexicons would be more likely to achieve successful communication, whereas words sampled from an atypical lexicon are less likely to be understood by other interlocutors, especially young children. The hypothesis, then, is that lexical typicality^1^ is shaped by communicative pressure, through a child’s understanding that other children possess limited lexicons. Conversely, atypical lexicons would suggest that communicative pressure is not yet present and that the individual either has immature social cognition skills or has not yet been exposed to other individuals with limited lexicons.

Using this latter technique, Mayor and Plunkett () measured lexical diversity in children aged 8–36 months. They reported that production of idiosyncratic language in production peaks at around 24 months. From this age, vocabularies tend to undergo a reduction in lexical diversity, so that individual lexicons overlap more at 30 months than at 24 months of age. As this non-monotonic trajectory is absent in receptive vocabularies, the authors hypothesized that communicative pressure might play a role in shaping productive vocabularies. The proposed scenario is as follows: for as long as infants and young children interact with adults, their productive vocabularies will be a subset of the adults’ lexicon, and all of their utterances will necessarily align with the receptive lexicon of the adult; there is no communicative pressure shaping their language production. However, when children interact with their peers, they need to select their utterances so that they are part of the receptive lexicon of others. In order to do so, they also need to understand that other children have limited lexicons. According to Mayor and Plunkett (2014), this ability emerges at approximately 24 months of age. Before this age, the lack of communicative pressure allows greater idiosyncrasy in their productive lexicons. Then, as children interact more with their peers, and become aware that other children have limited vocabularies, communicative pressure creates more stereotypical productive lexicons. No parallel pressure exists in perception, so receptive lexicons display a relatively even profile of idiosyncrasy throughout development (from about 15 months of age; see Mayor and Plunkett, 2011, 2014).

If this scenario is correct, we should expect the following hypotheses to be true:

(1) Children exposed to a larger number of other children should possess fewer lexical idiosyncrasies in production at 24 months of age than children exposed to a smaller number of other children. There are two ways in which children’s interactions with other children can affect lexical diversity. First, it has been found that the onset of Theory of Mind is typically earlier when children have siblings (Perner et al., 1994), and this may be true of social cognition skills in a broader sense. Second, children who have more opportunities to enact pretend roles with other children, will have more exposure to interlocutors with limited lexicons (Youngblade and Dunn, 1995). These two effects should work hand in hand to reduce lexical diversity in production at 24 months of age.
(2) Lexical idiosyncrasy in production should *not* be reduced with a greater number of children in the household at 18 months of age. An increase in overall lexical diversity over the next 6 months would suggest that communicative pressure has yet to act, as social cognition skills, such as level-I perspective taking (Moll and Tomasello, 2006) and cooperation skills (Brownell and Carriger, 1990) are still immature.
(3) The impact of household size on lexical idiosyncrasy in production should fade by 30 months of age. By this age, beyond natural variation in the development of social cognition, children have acquired social cognition skills that allow them to understand that other children have limited lexicons, independent of their individual exposure to differing numbers of other children.
(4) The level of lexical idiosyncrasy in comprehension should remain unaffected by changes in the learning environment throughout development. The effect of communicative pressure acts on production, not on comprehension.
(5) In addition, we expect to find gender effects, such as larger vocabularies in girls in comprehension and production (Eriksson et al., 2012).

The present contribution aims at testing the above-mentioned hypotheses using new participants, who were not part of the original sample used in Mayor and Plunkett (2014). To this end, communicative inventories were collected for 18-, 24-, and 30-month-old children in Mexico City, along with demographic information related to their families. Multiple regression analysis was used to identify the factors that modulated vocabulary size and lexical typicality in comprehension and production.

## Materials and Methods

### Participants

Families were recruited via advertising in the University Gazette and in a science museum, as well as in public transportation in Mexico City. Parental reports for a total of 550 young children were collected. All children were raised monolingual by Mexican Spanish-speaking parents. The age groups included: 153 18-month-olds (86 males, *M* = 17.9, *SD* = 0.5, range: 17.0–19.2), 153 24-month-olds (76 males, *M* = 24.0, *SD* = 0.9, range: 22.1–27.1), and 244 30-month-olds (141 males, *M* = 29.8, *SD* = 0.5, range: 27.2–32.1). All procedures performed in this study were in accordance with the ethical standards of the Mexican Psychology Society (Sociedad Mexicana de Psicología, Código ético del psicólogo, 2010). Before the experiment, written informed consent for study participation was obtained from all participants’ parents.

### Data Structure

A lexical inventory was adapted from the Oxford Communicative Development Inventory (Hamilton et al., 2000) for Mexican Spanish. This adaptation, the Inventario de Comprensión y Producción Lingüística en Infantes Mexicanos (hereafter referred to as the ICPLIM; Alva Canto and Hernández Padilla, 2001), was subsequently fined-tuned by analysing longitudinal recordings of Mexican children from 15 to 36 months of age engaged in a range of activities in day care centers: eating, playing, and interacting with their peers. Frequently used words were incorporated into the instrument (Alva Canto and Suárez, 2014). This method ensured the appropriateness of the instrument to index the vocabulary development of Mexican children. A recent comparison between production scores of the ICPLIM and the Mexican CDI (Jackson-Maldonado et al., 2003), based on the data of 176 children aged 12–30 months, yielded a correlation *r* = 0.97, *p* < 0.05 (Hernández Padilla and Alva Canto, 2014). The ICPLIM contains a total of 560 words divided into 19 semantic categories; it was chosen over the Mexican CDI as it allows for the assessment both of lexical production and comprehension.

For each age group, parents were asked to fill out the ICPLIM after they were given instructions by a trained research assistant (following guidelines reported in Jackson-Maldonado et al., 2003). Parents were also asked to provide the following demographic information: sex of the child, number of children in the household, number of siblings, number of adults living in the household, whether the child was attending day care (attendance for more than 2 months, at least ten hours per week) or not, parents’ ages and years of education (see **Table 1** for descriptive statistics). As can be seen in **Table 2**, there were strong correlations between individual factors (e.g., number of siblings and number of children in the household; the age of the mother and father). Consequently, the following factors were excluded from the regression analyses: number of siblings, and father’s age and years of education. The mother’s age, which was not expected to play a role in predicting language development, was excluded to reduce the risk of over-fitting the model. Simple regressions confirmed the absence of a role for these factors in accounting for variance in lexical size or lexical typicality (all *p*’s > 0.2).

**Table 1:** Descriptive statistics of the data.

	18 months			24 months			30 months		
	Mean	Min	Max	Mean	Min	Max	Mean	Min	Max
No. of siblings	0.46	0	3	0.47	0	3	0.54	0	4
No. of adults	3.07	1	12	3.04	1	8	3.00	1	9
No. of children	0.59	0	5	0.57	0	5	0.68	0	5
Age of mother	30.80	17	56	31.37	18	46	31.47	17	46
Mother’s education	14.50	6.5	20	14.86	6	25	14.82	6	22
Age of father	32.49	20	56	35.4	18	69	35.09	16	70
Father’s education	14.29	3	22	14.5	6	22	15.04	9	25
Norm. vocabulary (comp)	0.430	0.039	1.000	0.611	0.025	1.000	0.751	0.080	1.000
Norm. vocabulary (prod)	0.095	0.000	0.814	0.285	0.000	0.990	0.524	0.000	0.985
Lexical diversity (comp)	0.818	0.651	1.055	0.826	0.579	1.189	0.829	0.185	1.515
Lexical diversity (prod)	0.749	0.092	1.118	0.790	0.173	1.157	0.841	0.582	1.213

**Table 2:** Correlations at 18, 24, and 30 months of age.

	No. of siblings	No. of adults	No. of children	Age of mother	Mother’s education	Age of father	Father’s education
**18 months**							
No. of siblings	1						
No. of adults	-0.21*	1					
No. of children	0.83***	-0.07	1				
Age of mother	0.32***	-0.35***	0.30**	1			
Mother’s education	-0.29***	-0.05	-0.32***	0.12	1		
Age of father	0.31***	-0.31***	0.32***	0.84***	-0.01	1	
Father’s education	-0.28***	-0.14	-0.27***	0.15*	0.45***	0.22***	1
**24 months**							
No. of siblings	1						
No. of adults	-0.02	1					
No. of children	0.62***	0.23**	1				
Age of mother	0.22**	-0.24**	0.02	1			
Mother’s education	-0.27***	-0.11	-0.25**	0.31***	1		
Age of father	0.16*	-0.30***	0.00	0.71***	0.14	1	
Father’s education	-0.11	-0.20*	-0.11	0.35***	0.54***	0.27***	1
**30 months**							
No. of siblings	1						
No. of adults	-0.12	1					
No. of children	0.69***	0.13	1				
Age of mother	0.23**	-0.32***	0.03	1			
Mother’s education	-0.20**	-0.12	-0.21***	0.30***	1		
Age of father	0.12	-0.25***	-0.05	0.66***	0.12	1	
Father’s education	-0.09	-0.29***	-0.15*	0.30***	0.36***	0.36***	1

*^∗∗∗^*p* < 0.001, ^∗∗^*p* < 0.01, ^∗^*p* < 0.05*.

Lexical sizes were computed for all age groups, in comprehension and production, and were normalized to the total number of items on the ICPLIM lists before performing the regressions. Lexical typicality measures were obtained by computing the Euclidean distance between participants’ lexicons and the mean lexicon for their age group, where each word is either understood/produced (coded as 1 in a vector containing all words on the ICPLIM) or not under-stood/not produced (coded as 0). As this metric is strongly dependent on lexical size, the mean Euclidean distances are then normalized by the underlying binomial distribution, produced by measuring the Euclidean distance when vector values are drawn at random. This procedure ensures that lexical diversity and lexical size are independent. A detailed account of the method is described in Mayor and Plunkett (2014). The measurement thus obtained provides an account of lexical typicality; a large value indicates an atypical vocabulary and a low value a more “standard” vocabulary for a given age.

## Results

Multiple regression analyses were used to test whether the number of children in a household significantly predicted participants’ lexical size and typicality in production and comprehension. The following additional factors were considered in the multiple regression: Sex, Daycare attendance, and Mother’s years of education.

### 18 Months

#### Lexical Size in Comprehension and Production

No factor correlated with lexical size in comprehension or production.

#### Lexical Diversity in Comprehension

A series of regression analyses examined the relationships between lexical diversity in comprehension and Sex, Number of children in the household, Daycare attendance, and Mother’s years of education. **Table 3** shows the regression weights for the various models^2^. The full model had an *R*^2^ = 0.10, *F*(4, 145) = 3.934, *p* = 0.004, with Number of children and Mother’s years of education having significant regression weights. A reduced model, where Number of children and Mother’s years of education were included, had an *R*^2^ = 0.09, *F*(2, 147) = 7.016, *p* = 0.001. An *F*-test between the full model and the reduced model (comparing whether reduction in the residual sum of squares is statistically significant or not) revealed that the reduced model performed as well as the full model, *F*(2, 145) = 0.865, *p* = 0.42.

**Table 3:** Beta weights for the different regression models.

	18 months				24 months						30 months	
	Lexical diversity in comprehension		Lexical diversity in production		Lexical size in comprehension		Lexical size in production		Lexical diversity in production		Lexical size in production	
	Full	Reduced	Full	Reduced	Full	Reduced	Full	Reduced	Full	Reduced	Full	Reduced
Sex	0.010		0.024		-0.103^∗∗^	-0.097^∗∗^	-0.119^∗∗^	-0.113^∗∗^	-0.049^∗^		-0.078^∗^	-0.076^∗^
No. of children	0.017^∗∗∗^	0.018^∗∗∗^	0.045^∗^	0.024^∗^	-0.017		-0.029		-0.024^∗^	-0.022^∗^	-0.022	
Daycare	0.006		-0.028		-0.063		-0.050		-0.025		-0.020	
Mother’s education	0.004^∗^	0.004^∗^	0.004		0.004		0.000		0.000		0.002	

*^∗∗∗^*p* < 0.001, ^∗∗^*p* < 0.01, ^∗^*p* < 0.05*.

#### Lexical Diversity in Production

**FIGURE 1:**
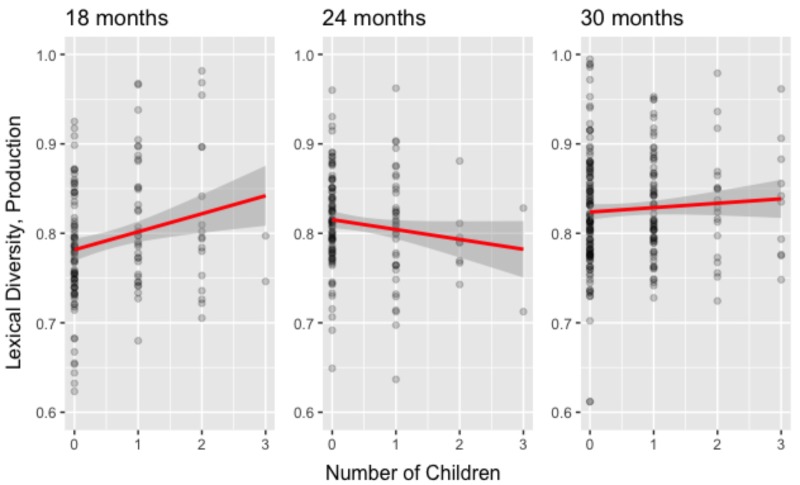
Lexical diversity in production as a function of the number of children in the household at 18, 24, and 30 months of age.

A similar series of regression analyses were performed to examine the relationships between lexical diversity in production and Sex, Number of children in the household, Daycare attendance, and Mother’s years of education. **Table 3** shows the regression weights for the various models. The full model had an *R*^2^ = 0.05, *F*(4, 145) = 1.875, *p* = 0.12, with Number of children carrying a significant regression weight. A reduced model, where Number of children was included, had an *R*^2^ = 0.03, *F*(1, 148) = 5.198, *p* = 0.024. This model performed as well as the full model, *F*(3, 145) = 0.775, *p* = 0.51. The left panel of **Figure 1** depicts how lexical diversity in production increases with the number of children in the household.

### 24 Months

#### Lexical Size in Comprehension

The full model had an *R*^2^ = 0.10, *F*(4, 144) = 3.846, *p* = 0.005, with Sex having significant regression weights (**Table 3**). A reduced model, where Sex was included, had an *R*^2^ = 0.06, *F*(1, 147) = 8.76, *p* = 0.004. This model performed as well as the full model, *F*(3, 144) = 2.139, *p* = 0.10. Boys had an average of 9.8% fewer words in their receptive lexicon than girls.

#### Lexical Size in Production

The full model had an *R*^2^ = 0.08, *F*(4, 144) = 3.138, *p* = 0.017, with Sex having significant regression weights. A reduced model, where Sex was included, had an *R*^2^ = 0.05, *F*(1, 147) = 8.17, *p* = 0.005. This model performed as well as the full model, *F*(3, 144) = 1.43, *p* = 0.23. Boys had an average of 11.3% fewer words in their receptive lexicon than girls.

#### Lexical Diversity in Comprehension

No factor correlated with lexical diversity in comprehension at 24 months of age.

#### Lexical Diversity in Production

The full model had an *R*^2^ = 0.06, *F*(4, 144) = 2.289, *p* = 0.006, with Sex and Number of children in the household having significant regression weights. A reduced model, where Number of children in the household was included,^3^ had an *R*^2^ = 0.03, *F*(1, 147) = 4.309, *p* = 0.040. This model performed as well as the full model, *F*(3, 144) = 1.598, *p* = 0.19. The middle panel of **Figure 1** depicts how lexical diversity in production decreases with the number of children in the household.

### 30 Months

#### Lexical Size in Comprehension

No factor correlated with lexical size in comprehension.

#### Lexical Size in Production

The full model had an *R*^2^ = 0.03, *F*(4, 229) = 1.568, *p* = 0.184, with Sex having significant regression weights (**Table 3**). A reduced model, where Sex was included, had an *R*^2^ = 0.018, *F*(1, 232) = 4.244, *p* = 0.041. This model performed as well as the full model, *F*(3, 229) = 0.682, *p* = 0.56. Boys had an average of 7.6% fewer words in their productive lexicon than girls.

#### Lexical Diversity in Comprehension and in Production

No factor correlated with lexical diversity in comprehension nor in production at 30 months of age.

## Discussion

Indices of lexical diversity were computed in both production and comprehension for 550 infants and toddlers. Lexical diversity in production at 24 months of age correlated *negatively* with the number of children in the household, supporting our first hypothesis: that increased exposure to young individuals provides experience of other interlocutors’ limited lexicons and the need to adjust the productive lexicon accordingly (in line with reports that interactions with other children help improve social cognition skills, e.g., Perner et al., 1994). We also observed the absence of a similar effect for lexical diversity in comprehension, which strengthened the communicative pressure hypothesis (our fourth hypothesis); no factors correlated with lexical diversity in comprehension at 24 months of age.

At 18 months of age, however, communicative pressure was not yet expected to act, as lexical diversity in production increases for another 6 months (Mayor and Plunkett, 2014) and socio-cognitive skills such as level-I perspective taking are still immature (Moll and Tomasello, 2006). This led to our second hypothesis, that the number of children in the household should not lead to a reduction in lexical diversity in production, as social cognitive skills are still too immature for children to understand that others may have limited lexicons. Not only did our results show no detrimental effect on lexical diversity with more children in the household, but they also revealed a *positive* correlation between the number of children in the household and lexical diversities in both production and comprehension. One potential explanation is that older children expose younger ones to a set of words that are typically not part of earlier lexicons. High frequency of exposure then leads to earlier acquisition (e.g., Barrett et al., 1991), and atypical words (for a young child) become part of the lexicon. The absence of communicative pressure at 18 months of age, due to immature social cognition skills, explains the symmetry of the effect; atypicality is larger, in both comprehension and production, with enhanced exposure to other children. It is noteworthy that the mother’s education correlates with lexical diversity in comprehension, but not in production, a result yet to be explained.

At 30 months of age, effects on lexical diversity in production, that were present at 24 months of age, are fading, in line with our third hypothesis. The impact of individual variation caused by living in households with more or fewer children is reduced, as children have acquired the set of socio-cognitive skills that allow them to interact with their peers. It is noteworthy that, in the present sample, lexical diversity in production is greater at 30 months of age than at 24 months, in contrast with the findings of Mayor and Plunkett (2014). Yet, closer inspection revealed that the 30-month olds in the present sample possess an average productive vocabulary score of 304 words, which matches the average productive vocabulary scores of the 24-month olds reported in Mayor and Plunkett (2014), and thus coincides with the peak of lexical diversity in production as reported in Mayor and Plunkett (2014).

Finally, regression analyses on vocabulary sizes revealed significant effects of sex at 24 months of age for both comprehension and production, and at 30 months of age for production. Girls were ahead of boys in their vocabulary development, in line with findings reported by other investigators (Eriksson et al., 2012), and as posited by our fifth hypothesis.

Our results add to a number of studies highlighting the relationships between social cognition and language (in particular for Theory of Mind: Harris et al., 2005; De Villiers, 2007). Several researchers have suggested that language is instrumental in shaping social cognition, by allowing complex representations of the thoughts of others (Segal, 1998; Hauser et al., 2002), and by providing children with the concepts to capture abstractions and mental states (Harris, 2005). Conversely, word learning typically takes place in a social situation, in which the naming event occurs when the infant and the speaker share attention (Tomasello and Farrar, 1986; Baldwin, 1994): Joint attention thus plays a foundational role as a precursor to social cognition in early language development.

The present study points to a bi-directionality of the interactions between language and social cognition and suggests a link between early environmental/social factors and social cognition development. Early exposure to their peers provides young children with an awareness of their limited language capacities, and this interaction may act as a catalyst for the maturation of social cognition (cf. Ruffman et al., 2002). These effects work together to shape their productive vocabulary.

In particular, it suggests the following scenario; socio-cognitive skills such as level-I perspective taking are still immature at 18 months of age (Moll and Tomasello, 2006) and hence do not allow toddlers to realize that their peers have limited lexicons. When relevant socio-cognitive skills emerge, at 24 months of age (Moll and Tomasello, 2006) they are modulated by the type of interactions with other children, similarly to Theory of Mind, being modulated by sibship size (Perner et al., 1994). Children who have more siblings tend to see their socio-cognitive skills mature earlier, in turn allowing them to fine-tune their productive lexicon to interact with their peers efficiently. At 30 months of age, children have seen their socio-cognitive skills mature sufficiently so that their productive lexicons are not modulated further by any imbalance in sibship size.

It may come as a surprise that the number of children in the household correlates with lexical diversity in production: in other words, that it is not merely a temporary adaptation to interaction with another, younger child, and that the process of fine-tuning utterances disappears when addressing adults, as has been shown for 4-year-old children (Shatz and Gelman, 1973). We can assume that the discovery of the limited lexicons of their peers does not provide children with evidence concerning the exact content of individual lexicons, and that an efficient strategy for communication is to use only words that are likely to be present in the receptive vocabularies of others. While at this point we can only speculate on the mechanisms children employ to decide which words are likely to be understood by others, a strategy of using words that are concrete, of high frequency, and of low phonological complexity is likely to be efficient (Braginsky et al., 2017).

Future research will need to take into consideration the ages of the other children living in the household, as the current study cannot evaluate the differential impact that older vs. younger children have on lexical typicality. Alternatively, systematic and direct measures of children’s interactions with their peers (potentially with a longitudinal design, as suggested by a reviewer) would help characterize further the environment individual children are raised in.

It should be acknowledged that the present study raises more questions than it answers, and that while the data is consistent with a hypothesis in which increased exposure to a number of other children affects social cognitive skills, and where communicative pressure shapes the productive lexicon, the amount of variance explained by predictors is small, and the data do not provide direct evidence for the bidirectional effect between social cognition and language acquisition. Nor do they address the process by which one has access to the content of another person’s lexicon: whether a first-person perspective (or simulation theory; e.g., Goldman, 2001), a third-person perspective (or Theory of Mind), or a second-person perspective (Przyrembel et al., 2012; Schilbach et al., 2013) is the most likely account for the observed phenomena. Future work should aim at directly assessing children’s social cognitive skills, particularly precursors of their Theory of Mind skills, so as to confirm directly what is, thus far, a conjecture.

## Author Contributions

JM and NA-T designed the study, collected and analyzed the data. All authors contributed to the manuscript, read and approved the submitted version.

## Conflict of Interest Statement

The authors declare that the research was conducted in the absence of any commercial or financial relationships that could be construed as a potential conflict of interest.

## Footnotes

1 In this manuscript, the terms “idiosyncratic knowledge,” “lexical overlap,” and “lexical typicality” are used interchangeably. An “atypical” vocabulary would contain many “idiosyncratic” words and “overlap” less with other lexicons, whereas a “typical” vocabulary would contain few “idiosyncratic” words and “overlap” more with other lexicons. There is greater “lexical diversity” where there are many “atypical” lexicons, and lesser diversity if many individual lexicons “overlap.”

2 For the sake of clarity, only significant model regressions are reported in **Table 1**.

3 Note that in the reduced model, Sex did not correlate with Lexical diversity in production at 24 months of age.
